# Fusion of the Genes *EWSR1* and *PBX3* in Retroperitoneal Leiomyoma with t(9;22)(q33;q12)

**DOI:** 10.1371/journal.pone.0124288

**Published:** 2015-04-14

**Authors:** Ioannis Panagopoulos, Ludmila Gorunova, Bodil Bjerkehagen, Sverre Heim

**Affiliations:** 1 Section for Cancer Cytogenetics, Institute for Cancer Genetics and Informatics, The Norwegian Radium Hospital, Oslo University Hospital, Oslo, Norway; 2 Centre for Cancer Biomedicine, Faculty of Medicine, University of Oslo, Oslo, Norway; 3 Department of Pathology, The Norwegian Radium Hospital, Oslo University Hospital, Oslo, Norway; 4 Faculty of Medicine, University of Oslo, Oslo, Norway; Tulane University School of Medicine, UNITED STATES

## Abstract

Retroperitoneal leiomyoma is a rare benign smooth muscle tumor almost exclusively found in women and with histopathological features similar to uterine leiomyomas. The pathogenesis of retroperitoneal leiomyoma is unclear and next to nothing is known about the cytogenetics and molecular genetics of the tumor. We present here a retroperitoneal leiomyoma with a t(9;22)(q33;q12) as the sole karyotypic aberration. The translocation resulted in an *EWSR1-PBX3* fusion gene in which exon 9 of *EWSR1* (nucleotide 1320 accession number NM_013986 version 3) was in-frame fused to exon 5 of *PBX3* (nucleotide 824 accession number NM_006195 version 5). The *EWSR1-PBX3* fusion transcript codes for a 529 amino acids long chimeric EWSR1-PBX3 protein which contains the N-terminal transactivation part of EWSR1 and the homeodomain of PBX3. The present study, together with our previous finding of a retroperitoneal leiomyoma with t(10;17)(q22;q21) as the sole karyotypic aberration and a *KAT6B-KANSL1* fusion gene, indicates that retroperitoneal leiomyomas may be characterized by fusion genes coding for chimeric proteins. However, cytogenetic and molecular heterogeneity exists in these tumors and it is too early to tell how many and which different pathways lead to retroperitoneal leiomyomagenesis.

## Introduction

Retroperitoneal leiomyoma is a rare benign smooth muscle tumor almost exclusively found in women [1–3]. Although anatomically distinct from their uterine counterparts, they have several pathological and histological features in common with uterine leiomyomas, including hyaline fibrosis, alternating myxoid change or trabecular patterns, and positivity for estrogen and progesterone receptors [1–3]. The cytogenetic and molecular genetic features of retroperitoneal leiomyomas remain largely unexplored as in the 2013 edition of “WHO classification of tumours of soft tissue and bone” there is no information about the genetics of these tumors [4]. Recently, we reported the first cytogenetically analyzed retroperitoneal leiomyoma which had a t(10;17)(q22;q21) as the sole karyotypic aberration [5]. Using RNA-sequencing we could demonstrate that the molecular consequence of the translocation was fusion of the *KAT6B* gene on 10q22 with the *KANSL1* gene (official full name: KAT8 regulatory NSL complex subunit 1) from 17q21 [5].

We present here the second cytogenetically analyzed retroperitoneal leiomyoma. The tumor cells were found to carry a t(9;22)(q33;q12) resulting in a fusion gene consisting of parts of *EWSR1* (from 22q12.2) and *PBX3* (from 9q33.3).

## Materials and Methods

### Ethics statement

The study was approved by the Regional Committee for Medical and Health Research Ethics, South-East Norway (REK Sør-Øst, Norge, http://helseforskning.etikkom.no), and written informed consent was obtained from the patient.

### Case history

**Fig 1:**
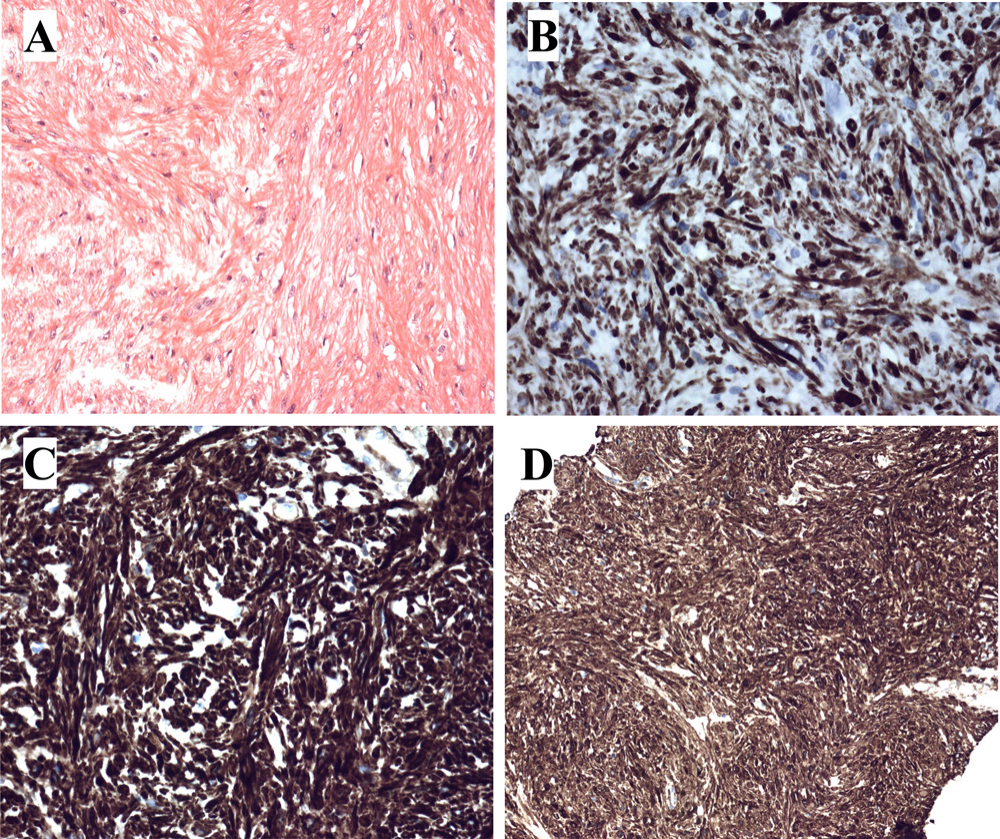
Histological examination of the retroperitoneal leiomyoma. A) HE-stained slide showing the tumor with spindle cells with eosinophilc cytoplasm without atypia surrounded by loose fibrous stroma. B) Immunoexpression of desmin. C) Immunoexpression of caldesmon. D) Immunoexpression of SMA.

A 26-year-old female presented with a slowly growing retroperitoneal tumor. A resection was performed and a 7.5 cm large tumor was removed. Microscopical examination showed fascicles of long spindle-shaped cells with eosinophilic cytoplasm surrounded by loose fibrous matrix (Fig 1A). Immunohistochemistry demonstrated positive staining for desmin (Fig 1B), actin, caldesmon (Fig 1C), SMA (Fig 1D), and CD99. Estrogen and progesterone receptors’ staining was weakly positive focally. There were negative findings for AE1/AE3, CD68, CD34, EMA, S-100, and CD31. Neither atypia nor necrosis was found. There were very few mitotic figures (0–1/10 high power field). The histological diagnosis was leiomyoma.

### G-banding and karyotyping

Fresh tissue from a representative area of the tumor was received and analyzed cytogenetically as part of our diagnostic routine. The sample was disaggregated mechanically and enzymatically with collagenase II (Worthington, Freehold, NJ, USA). The resulting cells were cultured and harvested using standard techniques. Chromosome preparations were G-banded with Wright stain and examined. The karyotype was written according to The International System for Human Cytogenetic Nomenclature (ISCN) 2009 guidelines [6].

### Fluorescence in situ hybridization (FISH)

FISH was performed on metaphase spreads using whole painting probes for chromosomes 9 and 22, a BCR/ABL(ABL1) Translocation Dual Fusion probe (Cytocell, Cambridge, UK), and an EWSR1 Breakapart probe (Cytocell, Cambridge, UK). Fluorescent signals were captured and analyzed using the CytoVision system (Leica Biosystems, Newcastle, UK).

### Molecular genetic analyses

Tumor tissue adjacent to that used for cytogenetic analysis and histologic examination had been frozen and stored at -80°C. Total RNA was extracted using Trizol reagent according to the manufacturer’s instructions (Lifetechnologies, Oslo, Norway) with the TissueLyser II homogenizer (Qiagen, Hilden, Germany).

Two μg of total RNA were reverse-transcribed in a 20 μL reaction volume using iScript Advanced cDNA Synthesis Kit for RT-qPCR according to the manufacturer’s instructions (Bio-Rad Laboratories, Oslo, Norway) and 1 μL was used as template in subsequent PCR assays. The 25 μL PCR volumes contained 12.5 μL of Premix Taq (Takara Bio Europe/SAS, Saint-Germain-en-Laye, France), 1 μL of cDNA, and 0.4 μM of each of the forward and reverse primers. The PCRs were run on a C-1000 Thermal cycler (Bio-Rad Laboratories). The PCR conditions for all amplifications were: an initial denaturation at 94°C for 30 sec followed by 35 cycles of 7 sec at 98°C and 2 min at 68°C, and a final extension for 5 min at 68°C. The quality of the cDNA synthesis was examined by amplification of a cDNA fragment of the *ABL1* gene using the primers ABL1-91F1 (5'-CAG CGG CCA GTA GCA TCT GAC TTT G-3') and ABL1-404R1 (5' CTC AGC AGA TAC TCA GCG GCA TTG C 3'). For the detection of the *EWSR1-PBX3* fusion transcript, two forward *EWSR1* primers, EWSR1-879F (5'- GCT ACC CCA TGC AGC CAG TCA CTG-3') and EWSR1-986F (5'- TAT GGG CAA CCG AGC AGC TAT GGA-3'), and two reverse PBX3 primers, PBX3-1198R1 (5'-GCT CCG ACT TGG GAC CCT TGG TAA G-3') and PBX3-1032R1 (5'-CAC GGC CGT CTT TGC AGC ATA GAG-3'), were used. The following primer combinations were applied: 1) EWSR1-879F together with the primer PBX1-1198R1, 2) EWSR1-986F together with the primer PBX3-1032R1, 3) EWSR1-879F together with the primer PBX3-1032R1, and 4) EWS-986F together with the primer PBX1-1198R1.

Three μL of the PCR products were stained with GelRed (Biotium), analyzed by electrophoresis through 1.0% agarose gel, and photographed. The remaining 22 μL of the PCR products were purified using the Qiagen PCR purification kit (Qiagen) and direct sequencing was performed using the light run sequencing service of GATC Biotech (http://www.gatc-biotech.com/en/sanger-services/lightrun-sequencing.html). The BLAST software (http://www.ncbi.nlm.nih.gov/BLAST/) was used for computer analysis of sequence data.

## Results

### G-banding and FISH

**Fig 2:**
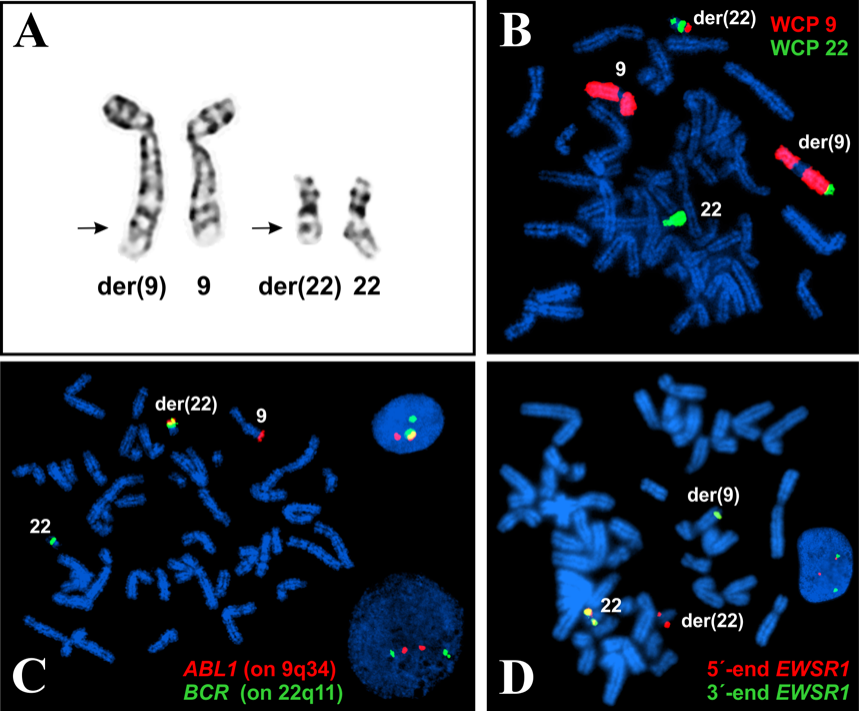
Cytogenetic and FISH data on the retroperitoneal leiomyoma. A) Partial karyotype showing the der(9)t(9;22)(q33;q12) and der(22)t(9;22)(q33;q12) with the corresponding normal chromosome homologs; breakpoint positions are indicated by arrows. B) FISH using whole chromosome painting probes for chromosome 9 (red signal) and 22 (green signal). C) FISH using the BCR/ABL(ABL1) Translocation Dual Fusion probe. D) FISH using the EWSR1 Breakapart probe.

The G-banding analysis yielded a karyotype with only a single chromosomal abnormality: 46,XX,t(9;22)(q33;q12)[10]/46,XX[5] (Fig 2A). FISH on metaphase spreads using painting probes for chromosomes 9 and 22 confirmed the 9;22-translocation (Fig 2B). To further characterize it, FISH was performed using a commercial probe for the fusion gene *BCR/ABL1* (Fig 2C). The analysis showed that the entire *ABL1* probe (red signal) was moved to der(22) where it generated a yellow fusion signal with the BCR probe which remained intact (green signal) on both the normal 22 and on the der(22)t(9;22). This indicated that the breakpoint on the der(9)t(9;22) was proximal to the *ABL1* locus whereas that on the der(22)t(9;22) was distal to the *BCR* locus. FISH analysis with the *EWSR1* break apart probe (Fig 2D) showed that the red, proximal part of the probe covering the 5´-end of the gene remained on the der(22)t(9;22) but that the green, distal part of the probe which hybridizes to the 3´-end of the *EWSR1* locus, had moved to the der(9)t(9;22). Thus the combined G-banding and FISH analyses suggested a rearrangement of *EWSR1* with possible fusion to a gene close but proximal to the *ABL1* locus in 9q34.12. We therefore searched for a fusion candidate gene up to 10 Mbp proximal to *ABL1* using the genome browser of UCSC Genome Bioinformatics (http://genome.ucsc.edu/index.html) and found the *PBX3* gene (in 9q33.3), 5 Mbp upstream of *ABL1*, which we deemed was the strongest candidate for further investigation as a 3´-partner in a *EWSR1*-fusion. This was because *PBX3* has extensive homology to *PBX1* [7] which has been found as a 3´-partner in an *EWSR1-PBX1* fusion in myoepithelial tumors [8, 9]. Furthermore, while we were busy analyzing the present retroperitoneal leiomyoma, an article reporting *EWSR1-PBX3* fusion in myopethelial tumors appeared on line [10], further supporting the hypothesis of *PBX3* as a potential fusion partner of *EWSR1*.

### Molecular genetic analysis

**Fig 3:**
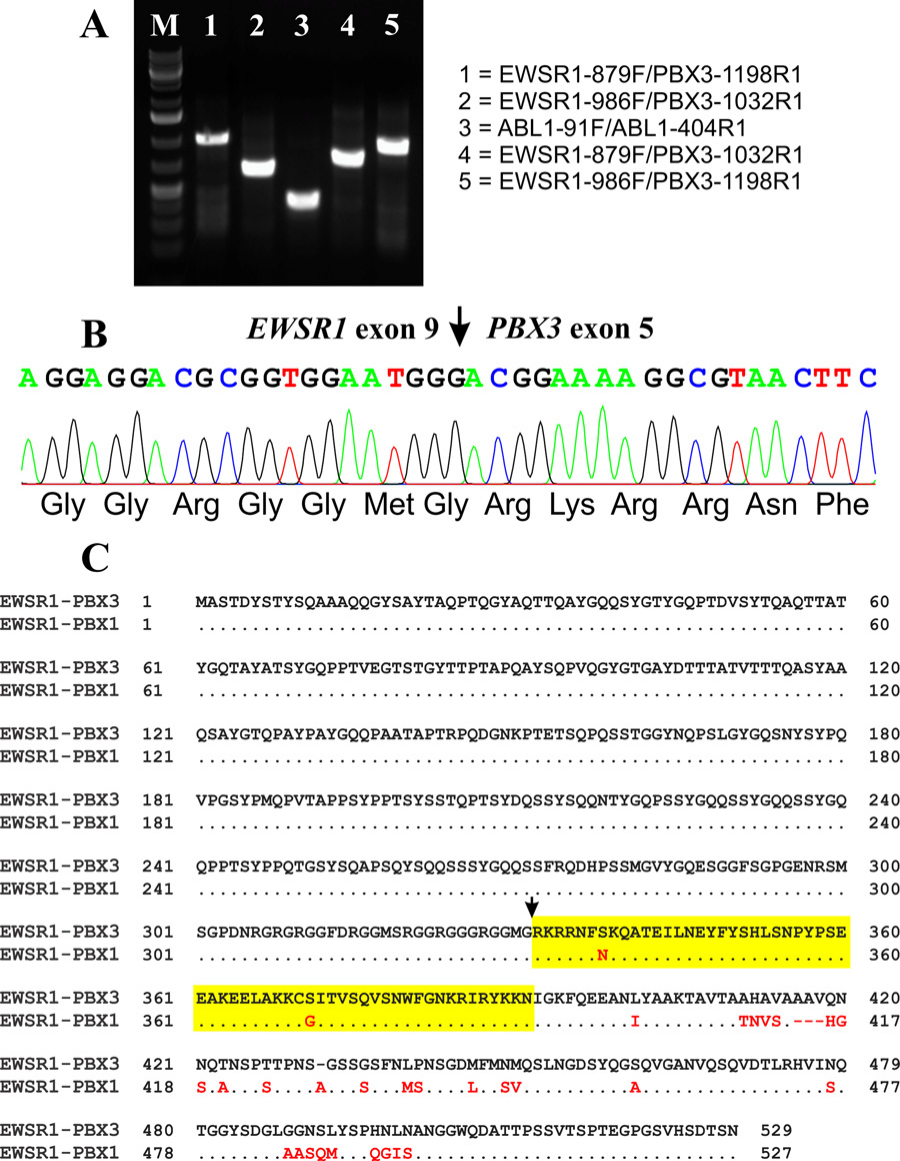
Molecular genetic data on the retroperitoneal leiomyoma. A) Gel electrophoresis of the RT-PCR amplified products using cDNA from the tumor. B) Partial sequence chromatogram of the amplified cDNA fragment showing the fusion point in the *EWSR1-PBX3* fusion transcript. C) Sequence alignment of the EWSR1-PBX3 protein and EWSR1-PBX1 protein. Homeodomains are shown in yellow color.

A 338 bp *ABL1* cDNA fragment was amplified indicating the good quality of the synthesized cDNA (Fig 3A). RT-PCR with all four primer combinations—EWSR1-879F/PBX3-1198R1, EWSR1-986F/PBX3-1032R1, EWSR1-879F/PBX3-1032R1, and EWSR1-986F/PBX3-1198R1—amplified cDNA fragments strongly suggesting the presence of an *EWSR1-PBX3* fusion transcript in the examined tumor (Fig 3A). Sequencing of the amplified cDNA fragment obtained with the EWSR1-879F/PBX3-1198R1 primer set showed that it was an *EWSR1-PBX3* chimeric cDNA fragment in which exon 9 of *EWSR1* (nucleotide 1320 accession number NM_013986 version 3) was in-frame fused to exon 5 of *PBX3* (nucleotide 824 accession number NM_006195 version 5) (Fig 3B).

## Discussion

We describe here the presence of an *EWSR1-PBX3* fusion gene brought about by a 9;22-translocation in a retroperitoneal leiomyoma. The case provides an additional example of involvement of the *EWSR1* gene in a benign tumor. Previously, *EWSR1* has been found to be pathogenetically rearranged in the benign hidradenoma of the skin, myoepithelioma, and hemangioma of bone [8, 9, 11, 12], in addition to its much more common involvement in malignant tumors such as the Ewing family of tumors, desmoplastic small round cell tumor, myxoid liposarcoma, and extraskeletal myxoid chondrosarcoma [13–16].

At the genomic level, the balanced 9;22-translocation we found as the sole chromosomal change is sufficient to generate a functional *EWSR1-PBX3* fusion, most probable on the derivative chromosome 22, since both *EWSR1* (in 22q12.2) and *PBX3* (in 9q33.3) are transcribed from centromere to telomere. The *EWSR1-PBX3* fusion gene described here is identical to the *EWSR1-PBX3* fusion found recently in three myopethelial tumors with low mitotic activity (0–1 per 10 high-power field) and immunohistochemically positive for S100 and EMA [10]. In both studies, exon 9 of *EWSR1* (former exon 8 in sequences with accession number X66899 and NM_005243; [17]) was fused to exon 5 of *PBX3*. *EWSR1-PBX3* is similar in this regard to other fusion genes involving *EWSR1*, such as *EWSR1-ATF1*, *EWSR1-PBX1*, and *EWSR1-ZNF444* [9, 18, 19]. Hence, *EWSR1* appears to have a mechanism of action in *EWSR1-PBX3* similar to that in the other *EWSR1*-involving fusions [20].

*PBX1* on 1q23.3, *PBX2* on 6p21.32, *PBX3* on 9q33.3, and *PBX4* on 19p13.11 code for the four members of the human PBX family of transcription factors [7, 21]. The *PBX* genes are known to regulate genes involved in the differentiation of urogenital organs and steroidogenesis through their ability to form hetero-oligomeric DNA complexes [22]. PBX proteins interact with a subset of HOX proteins resulting in increased DNA-binding affinity of HOX proteins thereby enhancing the transcription of downstream target genes [23]. In 1990, the prototype PBX1 transcription factor of the PBX family was identified based on its involvement in the t(1;19)(q23;p13) translocation seen most often in pre-B ALL [24, 25]. The t(1;19) generates the *TCF3-PBX1* fusion gene which codes for a chimeric protein where the N-terminal transactivation domain of TCF3 is fused to the C-terminal DNA binding homeodomain of PBX1. *PBX2*, *PBX3*, and *PBX4* were cloned as homologous to *PBX1* [7, 21]. The homeodomain of PBX3 shows 97% identity to the homeodomain of PBX1, 94% identity from amino acids 38 to 311, a region which includes the homeodomain, and 100% identity at the extreme C terminus. The overall identity between PBX1 and PBX3 is 84% [7]. In vitro experiments showed that PBX2 and PBX3 can be converted to oncoproteins by fusion with TCF3. Artificial TCF3-PBX2 and TCF3-PBX3 fusions, with the same part of TCF3 (amino acids 1 to 477) which is fused to PBX1 in t(1;19)-acute leukemias, displayed focus forming properties in NIH 3T3 fibroblasts comparable to those of the TCF3-PBX1 chimera, whereas none of the wild-type PBX2 and PBX3 proteins resulted in transformation of NIH 3T3 cells [26]. These results demonstrated that all three proteins, PBX1, PBX2, and PBX3, possess latent transforming properties that are similar in potency and dependence on fusion with TCF3 [26].

Information about the involvement of the *PBX3* gene in cancer is nevertheless limited. Recently, *PBX3* was found to be overexpressed in gastric cancer and to regulate cell proliferation [27]. In prostate cancer, expression of *PBX3* was found to be regulated by androgen and Let-7d [28]. In colorectal cancer, *PBX3* mRNA was negatively correlated with Let-7c levels so that *PBX3* could reverse the suppression growth effects of Let-7c [29]. The PBX3 protein was also found to be a critical cofactor of HOXA9 in leukemogenesis, and targeting of the interaction between the two proteins was deemed to be a feasible strategy to treat acute myeloid leukemia with *PBX3* gene overexpression [30].

Based on the *EWSR1* sequence with accession number NM_013986 version 3 and *PBX3* sequence with accession number NM_006195 version 5, the *EWSR1-PBX3* fusion transcript found here would be 3408 bp long and would code for 529 amino acids of chimeric EWSR1-PBX3 protein which contains the N-terminal transactivation part of the EWSR1 protein and the homeodomain of the PBX3 protein. In myoepitheliomas with t(1;22) and the *EWSR1-PBX1* fusion gene, two transcripts were reported: one in-frame and, hence, supposedly pathogenetically crucial, with fusion between exon 9 (exon 8 in sequences with accession number X66899 and NM_005243) of *EWSR1* and exon 5 of *PBX1*, the other out-of-frame with a fusion between exon 8 (exon 7 in sequences with accession number X66899 and NM_005243) of *EWSR1* and exon 5 of *PBX1*; this one is presumably pathogenetically unimportant [9]. The *EWSR1-PBX3* and the in-frame *EWSR1-PBX1* fusion transcripts code for proteins with high level of identity (Fig 3C) and, in principle, confirm the conclusions stemming from experiments with artificial TCF3-PBX2 and TCF3-PBX3 fusions [26].

The present study together with our previous work in which a retroperitoneal leiomyoma with a t(10;17)(q22;q21) as the sole karyotypic aberration was shown to have a *KAT6B*-*KANSL1* fusion gene, indicate that retroperitoneal leiomyomas may carry fusion genes coding for chimeric proteins. To what extent molecular heterogeneity exists in retroperitoneal leiomyomagenesis reflecting various pathogenetic pathways, is still largely unknown. Likewise, whether any pathogenetic differences exist between uterine and retroperitoneal leiomyomas remains to be studied more thoroughly.
